# Supplementary material for: Full Genome Nobecovirus Sequences From Malagasy Fruit Bats Define a Unique Evolutionary History for This Coronavirus Clade
Source: Front Public Health. 2022 Feb 11;10:786060. doi: 10.3389/fpubh.2022.786060 (PMC8873168; doi:10.3389/fpubh.2022.786060)
Supplement: Supplementary file 1 [file Data_Sheet_1.docx]

Supplementary Materials

Supplementary Figure 1. Read depth and coverage for full genome Nobecovirus sequences identified in Malagasy fruit bats.

Read depth after deduplication by CDHIT for full genome Madagascar fruit bat Nobecovirus contigs assembled in IDseq (78). Viral genomes were assembled from fecal specimens derived from one P. rufus (A) and two R. madagascariensis (B and C) bats.


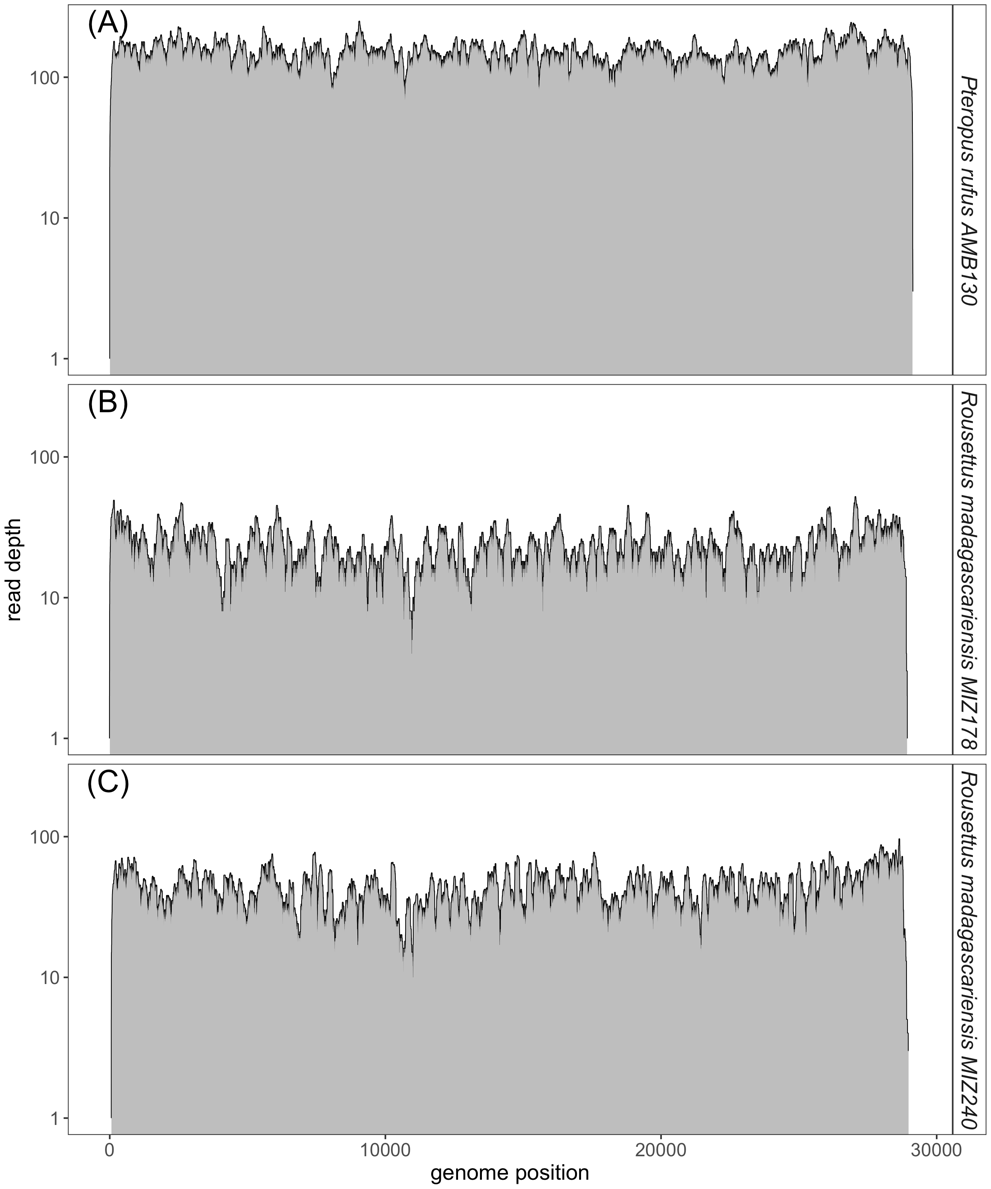


Supplementary Figure 2. ML amino acid Betacoronavirus phylogenies.

Maximum Likelihood amino acid phylogenies corresponding to translated sequences of the **(A)** spike, **(B)** envelope, **(C)** matrix, and **(D)** nucleocapsid *Betacoronavirus* proteins. All phylogenies were computed in RAxML-NG, using respective amino acid substitution models (A) WAG+I+G4+F, (B) LG+G4, (C) LG+I+G4, and (D) LG+I+G4+F (83,84). Bootstrap support values computed using Felsenstein’s method (85) are visualized on tree branches. In (A-D) novel Madagascar sequences are highlighted in yellow, and tip points are colored by *Betacoronavirus* subgenus, corresponding to the legend. Tip shape indicates whether the virus is derived from a bat (triangle) or non-bat (circle) host. Both trees are rooted in turkey *Gammacoronavirus,* accession number NC_010800. Branch lengths are scaled by amino acid substitutions per site, corresponding to the scale bar given indicated in each subplot.

**
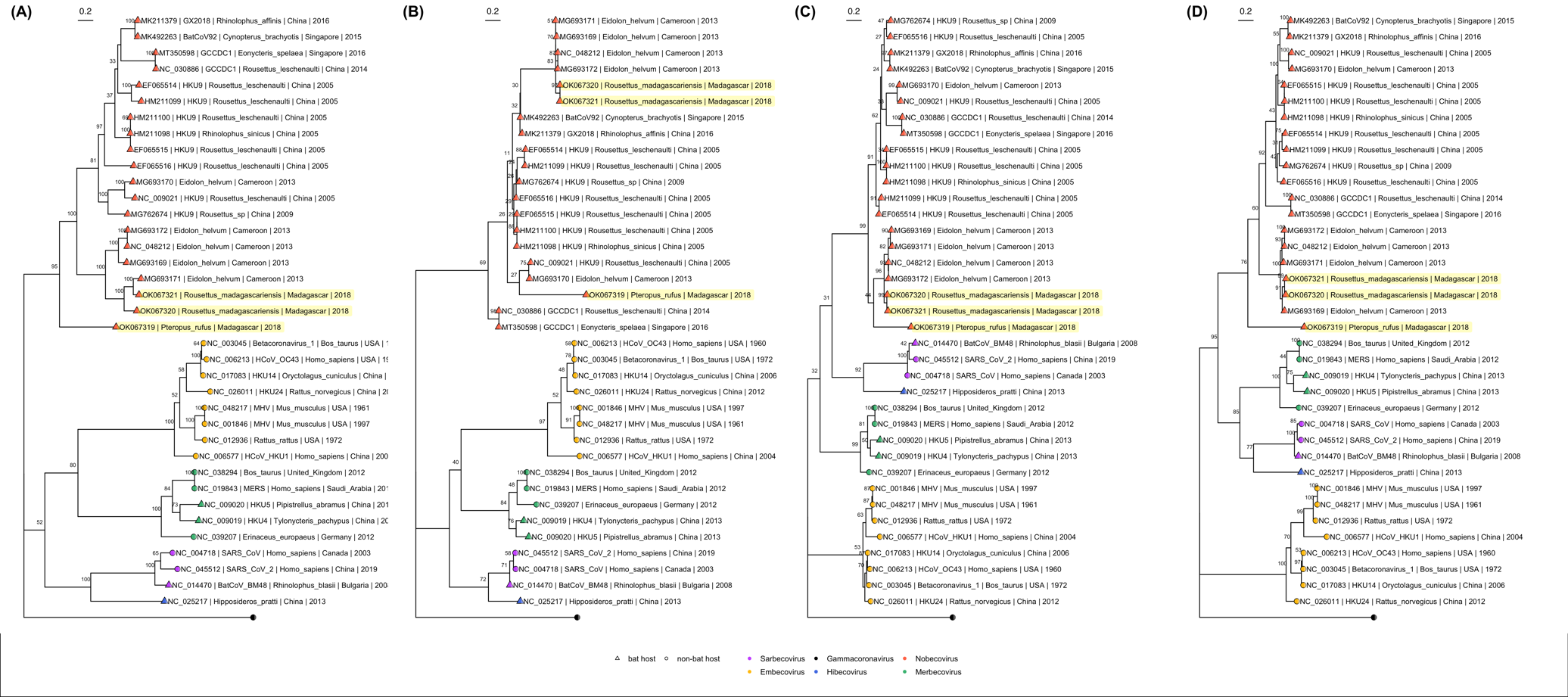
**

**Supplementary Figure 3: Bayesian phylogeny MRCA estimate of novel *P. rufus Nobecovirus***

Bayesian phylogeny to estimate time to MRCA for novel *P. rufus Nobecovirus* subclade. Plot depicts output of 600 million runs of a strict molecular clock Bayesian Skyline Coalescent model (GTR+I+G4) as implemented in BEAST2 (80,87). All major *Nobecovirus* subclades are depicted based on colored tip points, and the mean posterior estimates from averaging of all 600 billion trees after removal of 10% burn-in are visualized by color at the corresponding node. This figure includes the recombinant GCCDC1 lineage that was excluded in Figure 4 of the main text, resulting in broader HPD ranges at the most ancestral nodes. The dates of estimated time to MRCA for the *P. rufus Nobecovirus* subclade and the African Eidolon subclades are highlighted in red text.


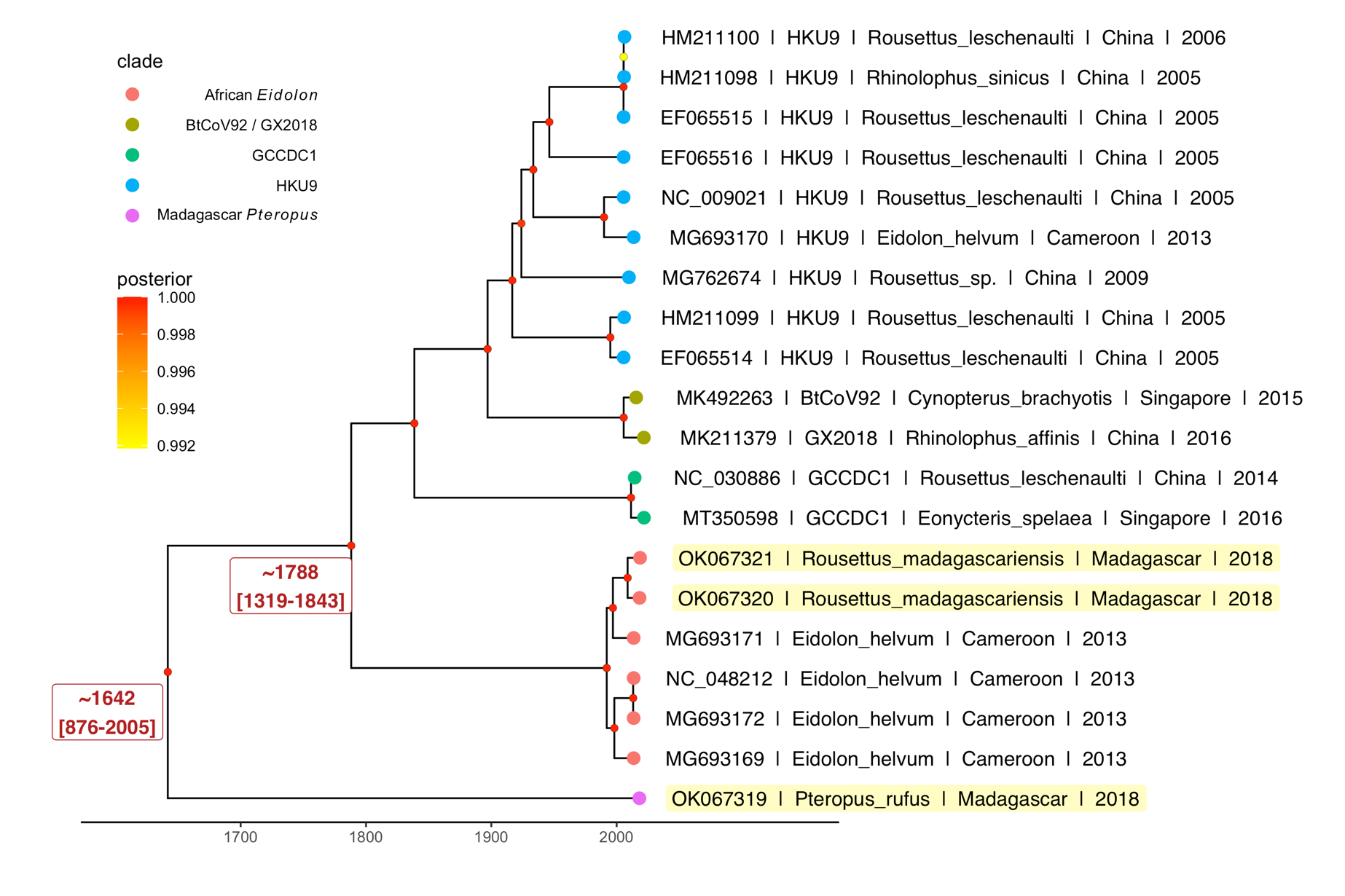


**Supplementary Table 1. Summary of BLAST queries to reference homologs for proteins identified in Malagasy fruit bat *Nobecoviruses.***

| **Virus accession number** | **Nucleotide or amino acid** | **β-HKU9**  **(MG762674)** | | **β--GCCDC1**  **(NC_030886)** | | **β-BtRt-BetaCoV/GX2018**  **(MK492263)** | | **β-Eidolon_helvum/Cameroon/2013**  **(NC_048212)** | |
| --- | --- | --- | --- | --- | --- | --- | --- | --- | --- |
|  |  | **% identity/query cover** | **Protein accession #** | **% identity/query cover** | **Protein accession #** | **% identity/query cover** | **Protein accession #** | **% identity/query cover** | **Protein accession #** |
| OK067319 | Genome* | 73.15/26* | MG762674 | 72.87/22* | NC_030886 | 73.35/26* | MK492263 | 73.54/26* | NC_048212 |
|  | ORF1a | 47.67/99 | AVP25405 | 53.7/98 | YP_009273004 | 54.52/98 | QEH60462 | 48.35/99 | YP_009824989 |
|  | ORF1b | 75.08/99 | AVP25405 | 75.2/99 | YP_009273004 | 72.72/99 | QEH60462 | 76.10/98 | YP_009824989 |
|  | S | 45.59/99 | AVP25406 | 47.3/99 | YP_009273005 | 46.93/99 | QEH60463 | 46.47/99 | YP_009824990 |
|  | NS3 | 40.38/72 | AVP25407 | 41.18/71 | YP_009273006 | 39.63/76 | QEH60464 | 41.67/73 | YP_009824991 |
|  | E | 50.68/92 | AVP25408 | 47.95/92 | YP_009273007 | 49.32/92 | QEH60465 | 46.58/92 | YP_009944266 |
|  | M | 58.56/99 | AVP25409 | 59.73/99 | YP_009273008 | 60.63/99 | QEH60466 | 62.44/99 | YP_009824992 |
|  | N | 50/89 | AVP25410 | 50.61/89 | YP_009273009 | 50.6/89 | QEH60467 | 48.27/89 | YP_009824993 |
|  | NS7a | --- | --- | --- | --- | --- | --- | --- | --- |
|  | NS7b | 40.28/98 | AVP25412 | --- | --- | 43.26/96 | QEH60470 | 40/99 | YP_009824995 |
| OK067320 | Genome* | 77/32* | MG762674 | 75.08/27* | NC_030886 | 75.95/34* | MK492263 | 95.15/89* | NC_048212 |
|  | ORF1a | 60.59/99 | AVP25405 | 59.36/99 | YP_009273004 | 60.22/99 | QEH60462 | 96.03/99 | YP_009824989 |
|  | ORF1b | 82.02/99 | AVP25405 | 81.09/99 | YP_009273004 | 82.36/99 | QEH60462 | 99.02/99 | YP_009824989 |
|  | S | 49.84/99 | AVP25406 | 50.86/98 | YP_009273005 | 50.62/99 | QEH60463 | 66.61/99 | YP_009824990 |
|  | NS3 | 46.93/75 | AVP25407 | 44.44/72 | YP_009273006 | 45.56/72 | QEH60464 | 89.32/99 | YP_009824991 |
|  | E | 58.11/97 | AVP25408 | 55.41/97 | YP_009273007 | 66.22/97 | QEH60465 | 92/98 | YP_009944266 |
|  | M | 70.70/99 | AVP25409 | 70.75/99 | YP_009273008 | 70.75/99 | QEH60466 | 91.98/99 | YP_009824992 |
|  | N | 61.19/98 | AVP25410 | 59.25/96 | YP_009273009 | 62.80/98 | QEH60467 | 86.6/99 | YP_009824993 |
|  | NS7a | 55.65/76 | AVP25411 | 25.89/57 | YP_009273011 | 54.78/76 | QEH60469 | --- | --- |
|  | NS7b | 80/95 | AVP25411 | --- | --- | 77.14/95 | QEH60469 | --- | --- |
|  | NS7c | 46.1/96 | AVP25412 | 28.57/82 | YP_009273013 | 47.14/95 | QEH60470 | 45.89/99 | YP_009824995 |
| OK067321 | Genome* | 77.06/32 | MG762674 | 75.25/30 | NC_030886 | 75.99/33 | MK492263 | 95.31/89 | NC_048212 |
|  | ORF1a | 60.54/99 | AVP25405 | 59.41/99 | YP_009273004 | 60.09/99 | QEH60462 | 96.15/99 | YP_009824989 |
|  | ORF1b | 81.91/99 | AVP25405 | 81.12/99 | YP_009273004 | 82.13/99 | QEH60462 | 99.13/99 | YP_009824989 |
|  | S | 51.05/99 | AVP25406 | 50.6/96 | YP_009273005 | 50.31/99 | QEH60463 | 66.61/99 | YP_009824990 |
|  | NS3 | 45.81/75 | AVP25407 | 44.44/72 | YP_009273006 | 44.97/72 | QEH60464 | 89.74/99 | YP_009824991 |
|  | E | 58.11/97 | AVP25408 | 55.41/97 | YP_009273007 | 66.22/97 | QEH60465 | 92/98 | YP_009944266 |
|  | M | 71.43/97 | AVP25409 | 71.23/99 | YP_009273008 | 71.23/99 | QEH60466 | 91.98/99 | YP_009824992 |
|  | N | 61.62/98 | AVP25410 | 61.73/86 | YP_009273009 | 67.48/87 | QEH60467 | 87.23/99 | YP_009824993 |
|  | NS7a | 53.28/96 | AVP25411 | --- | --- | 52.76/88 | QEH60469 | --- | --- |
|  | NS7b | 66.67/92 | AVP25411 | --- | --- | 66.67/92 | QEH60469 | 42.86/35 | YP_009824995 |
|  | NS7c | 46.81/96 | AVP25412 | 28.57/82 | YP_009273013 | 47.86/95 | QEH60470 | 45.89/99 | YP_009824995 |

*BLASTn query indicated by superscript. All other queries were BLASTx.

---BLASTx query generated no hits.

NOTE: ORF1b was BLASTx queried as the segment of ORF1ab that did not overlap ORF1a.

**Supplementary Table 2. Summary of BLAST queries to reference homologs for proteins identified in Malagasy fruit bat *Nobecoviruses.***

| **Virus accession number** | **Nucleotide or amino acid** | **Top BLAST hit** | **% identity of top BLAST hit** | **% coverage of top BLAST hit** | **Accession number of top BLAST hit** |
| --- | --- | --- | --- | --- | --- |
| OK067319 | Genome* | Bat coronavirus HKU9, complete genome | 73.64 | 27 | EF065514 |
|  | ORF1a | ORF1a [Bat coronavirus] | 48.36 | 99 | AWV67046 |
|  | ORF1b | ORF1ab polyprotein [Bat coronavirus] | 76.1 | 98 | YP_009824989 |
|  | S | spike glycoprotein [Bat coronavirus] | 46.93 | 99 | QEH60463 |
|  | NS3 | hypothetical protein [Bat coronavirus HKU9] | 42.07 | 76 | ADM33567 |
|  | E | envelope protein [Eidolon bat coronavirus/Kenya/KY24/2006 | 46.58 | 92 | ADX59468 |
|  | M | Membrane glycoprotein [Bat coronavirus HKU9] | 63.13 | 97 | ABN10930 |
|  | N | N protein [Rousettus bat coronavirus HKU9] | 52.25 | 88 | AVP25400 |
|  | NS7a | Low-density lipoprotein receptor-related protein 1-like [Rhinipicephalus sanguineus] | 31.25 | 40 | XP_037511191 |
|  | NS7b | NS7b [Rousettus bat coronavirus HKU9] | 40.28 | 98 | AVP25412 |
| OK067320 | Genome* | Bat coronavirus isolate CMR900 | 95.24 | 94 | MG693169 |
|  | ORF1a | ORF1a [Bat coronavirus] | 96.41 | 99 | AWV67062 |
|  | ORF1b | ORF1ab polyprotein [Bat coronavirus] | 99.02 | 99 | YP_009824989 |
|  | S | Spike protein [Bat coronavirus] | 76.75 | 99 | AWV67064 |
|  | NS3 | ORF3 protein [Eidolon bat coronavirus Kenya/KY24/2006] | 94.54 | 99 | ADX59467 |
|  | E | envelope protein [Eidolon bat coronavirus/Kenya/KY24/2006] | 94.67 | 98 | ADX59468 |
|  | M | membrane protein [Bat coronavirus] | 91.98 | 99 | YP_009824992 |
|  | N | capsid [Bat coronavirus] | 91.03 | 99 | AWV67051 |
|  | NS7a | hypothetical protein ORFx [Bat coronavirus] | 60.29 | 90 | AWV67068 |
|  | NS7b | hypothetical protein [Bat coronavirus HKU9] | 88.57 | 95 | ADM33571 |
|  | NS7c | hypothetical protein ORFy [Eidolon bat coronavirus/Kenya/KY24/2006] | 91.61 | 99 | ADX59472 |
| OK067321 | Genome* | Bat coronavirus isolate CMR891-892 | 92.6 | 99 | MG693171 |
|  | ORF1a | ORF1a [Bat coronavirus] | 96.5 | 99 | AWV67062 |
|  | ORF1b | ORF1ab polyprotein [Bat coronavirus] | 99.13 | 99 | YP_009824989 |
|  | S | Spike protein [Bat coronavirus] | 86.94 | 99 | AWV67064 |
|  | NS3 | ORF3 [Bat coronavirus] | 95.3 | 99 | AWV67065 |
|  | E | envelope protein [Eidolon bat coronavirus/Kenya/KY24/2006] | 94.67 | 98 | ADX59468 |
|  | M | membrane protein [Bat coronavirus] | 91.98 | 99 | YP_009824992 |
|  | N | nucleocapsid protein [Eidolon bat coronavirus/Kenya/KY24/2006] | 91.22 | 99 | ADX59470 |
|  | NS7a | hypothetical protein ORFx [Bat coronavirus] | 80 | 91 | AWV67068 |
|  | NS7b | hypothetical protein [Bat coronavirus HKU9] | 72.22 | 92 | ADM33571 |
|  | NS7c | hypothetical protein ORFy [Eidolon bat coronavirus/Kenya/KY24/2006] | 91.61 | 99 | ADX59472 |

*BLASTn query indicated by superscript. All other queries were BLASTx. ORF1b was queried as the part of ORF1ab excluding ORF1a.
